# Effects of Mung Bean (*Vigna radiata*) Protein Isolate on Rheological, Textural, and Structural Properties of Native Corn Starch

**DOI:** 10.3390/polym14153012

**Published:** 2022-07-25

**Authors:** Mohammad Tarahi, Sara Hedayati, Fakhri Shahidi

**Affiliations:** 1 Department of Food Science and Technology, Faculty of Agriculture, Ferdowsi University of Mashhad (FUM), Mashhad 9177948978, Iran; tarahimohammad@yahoo.com; 2 Nutrition Research Center, School of Nutrition and Food Sciences, Shiraz University of Medical Sciences, Shiraz 7193635899, Iran

**Keywords:** corn starch, mung bean protein isolate, rheology, texture, syneresis

## Abstract

It is critical to understand the starch–protein interactions in food systems to obtain products with desired functional properties. This study aimed to investigate the influence of mung bean protein isolate (MBPI) on the rheological, textural, and structural properties of native corn starch (NCS) and their possible interactions during gelatinization. The dynamic rheological measurements showed a decrease in the storage modulus (G’) and loss modulus (G”) and an increase in the loss factor (tan δ), by adding MBPI to NCS gels. In addition, the textural properties represented a reduction in firmness after the addition of MBPI. The Scanning electron microscope (SEM) images of the freeze-dried NCS/MBPI gels confirmed that the NCS gel became softer by incorporating the MBPI. Moreover, X-ray diffraction (XRD) patterns showed a peak at 17.4°, and the relative crystallinity decreased with increasing MBPI concentrations. The turbidity determination after 120 h refrigerated storage showed that the addition of MBPI could reduce the retrogradation of NCS gels by interacting with leached amylose. Additionally, the syneresis of NCS/MBPI gels decreased at 14 days of refrigerated storage from 60.53 to 47.87%.

## 1. Introduction

Starch and protein are two abundant and nutritionally important constituents of the human diet that have different physicochemical and functional characteristics and play an important role in the quality and nutritional properties of food products [1,2,3]. Starch is a tasteless and odorless polysaccharide that is cultivated to produce more than 50 million tons/year globally. It has a semi-crystalline structure that is typically made of amylose (70–85%) and amylopectin (15–30%) macromolecules [4,5,6]. Starch is widely utilized in many food and non-food industries, due to its low-cost, easy availability, biodegradability, and non-toxicity characteristics. Moreover, it is the main component of starchy foods, such as pasta, bread, and noodle [3,7]. However, native starch has some limitations that restrict its application in the food industry, such as high retrogradation tendency and poor shear and thermal stability [8]. Moreover, protein-energy malnutrition is becoming a serious issue, especially in developing countries, due to insufficient protein intake and poor food quality [9]. To meet the consumer demands for improving the textural and the nutritional (high-protein foods) quality of the products, some proteins from different sources (cereals, legumes, milk, meat, and fish), as a safe supplement, have been added to the starch-based foods [1,3,10]. Therefore, proteins have technological and nutritional roles in high-protein starchy products [11].

The second-highest ingredient in most of the starchy foods is typically protein (4–20% *w*/*w*) that is naturally present in the food matrix or added to improve the physicochemical properties of the product [1,3,12]. Proteins are important components in the human diet, due to their essential roles in immune responses, repairing damaged cells, and muscle mass maintenance [13]. Moreover, the proteins can play as texture- and structure-macromolecular modifiers because of their aggregation and gel formation during the process or their interactions with the other biopolymers in the food matrix, such as starch, which leads to the improvement of the texture stability of the final product [10,11]. Animal and plant proteins are two primary sources of proteins in the human diet. Recent research has revealed the disadvantages of high animal protein intake, such as high blood pressure, coronary heart disease, and obesity. On the contrary, the consumption of plant proteins is highly recommended due to their health benefits, such as anti-cancer activities, anti-cardiovascular disease, reduced glycemic index, and weight management [13,14].

Plant proteins are currently widely utilized in the food industry, mainly from legumes, due to their excellent functional characteristics, such as foam formation, emulsification, gelation, solubility, film-forming, water holding capacity (WHC), and the increase in consumers’ demand for plant-based foods [14]. The WHC of protein is related to protein–water interactions that can affect the water distribution in the food matrix and modify the interactions of water and other components, which is a crucial property in starch-based food. Moreover, proteins with high WHC can hold water in the system more efficiently, which leads to better structural characteristics [3]. Mung bean protein isolate (MBPI) has recently attracted the attention of many food industry researchers, due to its desirable functions, including foaming, emulsification, and WHC. Previous studies have shown that the MBPI has higher WHC than the commercial soy protein isolate, fenugreek protein concentrate, chickpea protein isolate, and safflower protein isolate, due to its high phosphate and other polar groups [15,16]. Therefore, the high WHC of MBPI may be the favorable property for its application in starch-based products.

The interactions between starch and protein have been increasingly taken into consideration during the last few years, for instance, the interactions between starch and potato [17], zein, soy, and whey [18], pea, rice, egg albumin, and whey [11], lentil [19], and peanut [20] proteins. It is critical to understand the starch and protein interactions and their behavior during gelatinization to obtain the food products with desired rheological, textural, and structural characteristics. However, different starch-protein systems could lead to varying characteristics of the final product and contradictory results have been reported by different researchers. For example, Yang et al. [12] reported that the G’ and G’’ of corn starch decreased after the addition of whey protein isolate, which indicates the weakening of the gel network, while Qiu et al. [21] demonstrated that the addition of soy protein isolate could increase the G’ and G’’ of the corn starch and waxy corn starch. Therefore, this study aimed to investigate the influence of MBPI on the rheological, textural, and structural properties of native corn starch (NCS) and the NCS–MBPI interactions.

## 2. Materials and Methods

### 2.1. Materials

NCS was kindly donated by the Behinazma company (Shiraz, Iran). Mung bean seeds were obtained locally (Shiraz, Iran). Mung bean seeds were cleaned, washed, and milled into flour using an electrical miller (model OE-830, Olympia, Glarus, Switzerland). All chemical materials were of analytical grade and were supplied by Sigma Chemical Co. (St Louis, MO, USA).

### 2.2. Preparation of MBPI

Protein isolate was extracted according to the method described by El-Adawy [22] with slight modifications. Briefly, the dispersion of mung bean flour in distilled water (1:10 *w*/*v*) was adjusted to pH 9 with 1 N NaOH and stirred for 1 h. Then, the dispersion was centrifuged (3000× *g*, 6 min). The supernatant was adjusted to pH 4.5 with 1 N HCl and centrifuged (3000× *g*, 6 min). The precipitated protein was washed two times, neutralized with 0.1 N NaOH, and freeze-dried. The protein was 89%, as determined by the micro-Kjeldahl method [23].

### 2.3. Preparation of NCS/MBPI Blends

Different concentrations (0, 2, 4, 6 and 8%) of MBPI was added to NCS and mixed using a mixer (PARS KHAZAR, BG-300P).

### 2.4. Dynamic Rheological Properties

NCS/MBPI slurry (6%, *w*/*w*, dry basis) was gelatinized in a water bath for 30 min at 95 °C and then cooled to 25 °C. The viscoelastic behavior of the NCS/MBPI gels was determined with a rheometer (MCR-302, Anton Paar, Graz, Austria), equipped with a cone (1° cone angle) and plate geometry (1 mm gap and 25 mm cone diameter) at 25 °C. The dynamic rheological properties, storage modulus (G’), loss modulus (G”) and loss factor (tan δ = G”/G’) were recorded in the linear viscoelastic region with a frequency sweep range of 0 rad/s to 62.83 rad/s (0.01 to 10 Hz) at 1.0% strain.

### 2.5. Textural Properties

The NCS/MBPI gels were prepared according to a previous test (Section 2.4) and placed in plexiglass holders (20 mm diameter and 10 mm height), then covered and held at 4 °C overnight. The textural properties of gels were analyzed using a TA-XT texture analyzer (Stable Micro Systems, Surrey, England), as described by Hedayati and Niakousari [24]. The gels were compressed with a 40-mm flat probe at the speed of 1 mm/s and the deformation level of 25%.

### 2.6. Morphological Properties

Gels were prepared as described in Section 2.5 and then freeze-dried. The morphological properties of the freeze-dried gels were observed by SEM (TESCAN-Vega 3, Brno, Czech Republic). The cross-section of samples was fixed on SEM stub and coated with gold using a sputter coater (Q150R-ES, Quorum Technologies Ltd, Lewes, UK). The SEM micrographs were taken at an accelerating voltage of 20 kV and a 300× magnification.

### 2.7. X–ray Diffraction (XRD) Analysis

The freeze-dried samples were prepared according to Section 2.6, and the XRD experiment was performed using an X-ray diffractometer (D8-Advance, Bruker, Karlsruhe, Germany) at 40 kV and 40 mA. The diffraction angle was 5 to 30°, and the scanning speed of 4°/min. The relative crystallinity (RC) of the samples was evaluated by the software (OriginPro v9.8.0.200, Thermo Fisher Scientific Inc., Waltham, MA, USA).

### 2.8. Turbidity Measurements

Turbidity of different gel samples was evaluated as described by Singh et al. [25]. Briefly, 1% aqueous suspensions of NCS/MBPI blends (Section 2.3) were heated for 30 min in a water bath (90 °C). The samples were stored at 4 °C and the turbidity was measured every 24 h at 640 nm using a double beam UV–vis spectrophotometer (Halo DB-20R, Dynamica, Livingston, UK).

### 2.9. Syneresis

The syneresis of the NCS/MBPI blends was determined by the method of Shams-Abadi and Razavi [8] with some modifications. Next, 10 g of NCS/MBPI gels (4%) were prepared in 50 mL centrifuge tubes, as described before. Then, the centrifuge tubes were stored for 1, 7, and 14 days at 4 °C. The samples were centrifuged (Sigma 3–30 KS, Osterode am Harz, Germany) at 8000× *g* for 5 min. The syneresis of each sample was measured by the following Equation (1):

Syneresis = supernatant weight/initial sample weight × 100(1)

### 2.10. Statistical Analyses

All experiments were performed in triplicate. Analysis of variance (ANOVA) was performed (*p* < 0.05) between means using the Duncan’s multiple range test by SPSS software, version 26 (IBM Company, Chicago, IL, USA).

## 3. Results and Discussion

### 3.1. Dynamic Rheological Properties

**Figure 1:**
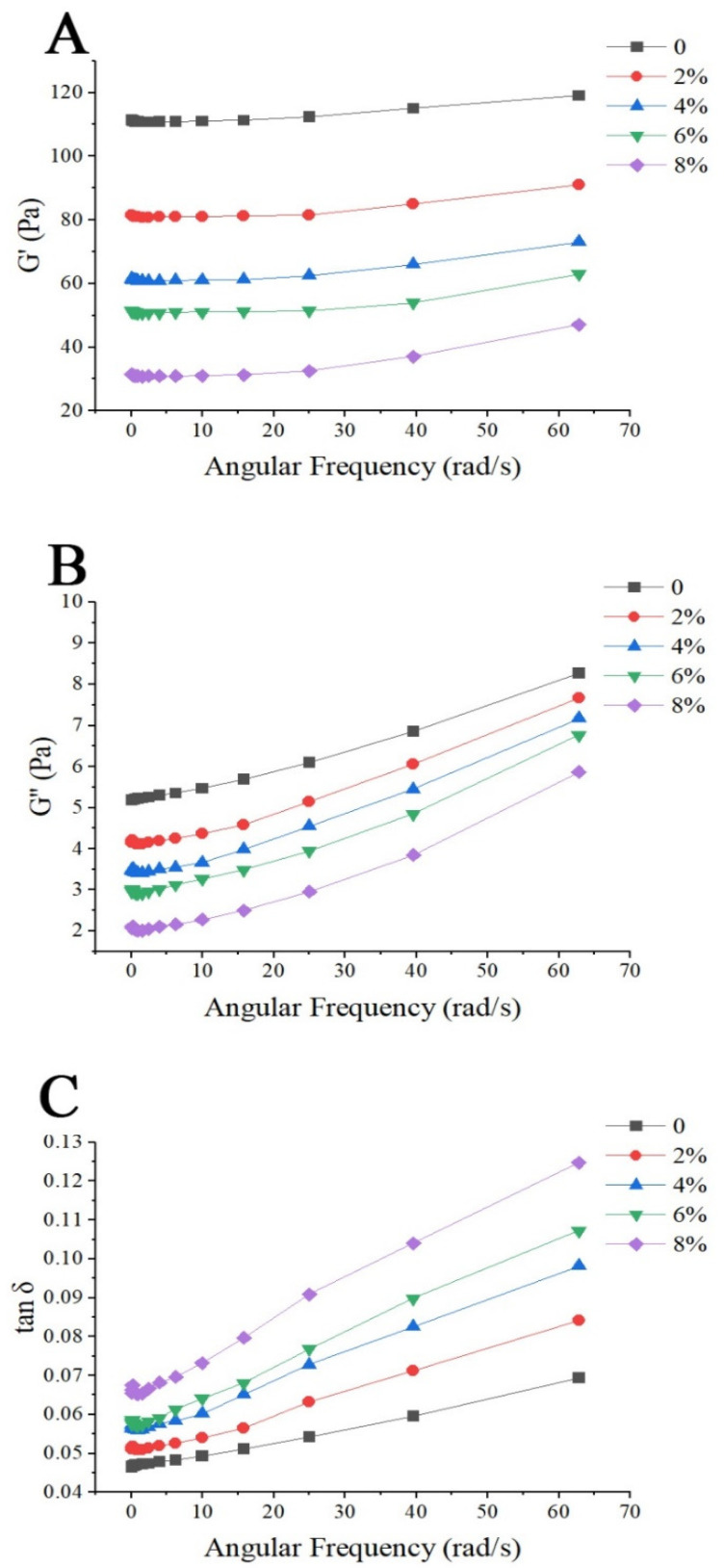
Dynamic rheology of NCS/MBPI gels: (**A**) storage modulus, (**B**) loss modulus, and (**C**) loss factor.

Dynamic modulus is a suitable tool for studying the interactions between dispersed and continuous phases in polymer solutions, which plays an important role in the sensory and quality evaluation of starch-based products [3,26]. The changes in storage modulus (G’), loss modulus (G”), and loss factor (tan δ) in the frequency range of 0.01–10 Hz are shown in Figure 1. By adding MBPI to NCS, G’ and G” of the system decreased, and tan δ increased, which indicates the weakening of the gels’ network [27]. In addition, with an increase in frequency, all rheological parameters (G’, G” and tan δ) increased, indicating a weak gel network of NCS with/or without the addition of MBPI. However, by adding MBPI, the NCS/MBPI gels presented a higher sensitivity to frequency changes, indicating the weaker gel structure of NCS/MBPI than the NCS gel [26,27]. In addition, all gel samples had a tan δ less than 1 (Figure 1C). This phenomenon indicates the solid (viscoelastic) behavior of gels throughout the frequency region, which increased with increasing MBPI concentration, and the viscoelastic behavior of the gels shifted from solid-like to liquid-like [12,19]. Previously, Sang et al. [28] reported that adding a small amount of ovalbumin to wheat dough could reduce the dough’s G’ and G’’, but increase the tan δ. They suggested that the ovalbumin might serve as a lubricating agent in doughs so that the increasing ovalbumin content makes doughs softer and less elastic. Zhou et al. [27] demonstrated that by increasing the whey protein ratio in wheat doughs, the G’ and G’’ decreased and tan δ increased. However, an opposite trend was observed by the increasing soy protein isolate ratio in the system, which may be due to the aggregation of soy protein isolate and increase in disulfide bonds in the system. Furthermore, Niu et al. [29] suggested that the reduction in G’ after adding protein may be due to its interactions, especially the interactions of carboxyl groups of amino acids with leached amylose, which inhibited the amylose gelation and reduced its G’. Moreover, Yang et al. [12] reported that whey protein isolate could act as an inactive filler in corn starch gel and prevent amylose rearrangement, which decreases the elastic modulus (G’ and G”) and weakenes the corn starch gel network. These observations suggest that the MBPI could weaken the gel network of NCS by acting as a lubricant and inactive filler in the NCS/MBPI system because of its high WHC or/and interact with leached amylose during gelatinization via its carboxyl groups and retard the amylose rearrangement.

### 3.2. Textural Properties

The effects of MBPI on the textural properties (hardness, cohesiveness, springiness, and gumminess) of NCS gels are shown in Table 1. The hardness of the gel samples displays gel strength under compression, which indicates the physical characteristics of foodstuffs [30]. The hardness of NCS/MBPI gels was significantly decreased by increasing MBPI concentrates (*p* < 0.05). Sun and Xiang [20] and Kumar et al. [31] showed the reduction in the gel hardness of starch-protein compositions after substituting the starch with the protein, which could be due to the decrease in amylose content. Because the amylose content of the system plays an important role in hardness determination, and the reduction in amylose content causes lower firmness and weakens the gel structure [19,32]. In addition, Liu et al. [33] reported that the gel firmness is primarily influenced by leached amylose rearrangement rather than amylopectin retrogradation. In the present study, the starch content of the system was constant, but the gel hardness decreased after the MBPI addition. This phenomenon indicates the interaction of MBPI with leached amylose during the gelatinization and disturbing the cross-linking interactions between starch molecules. The same results for starch-protein systems have been reported previously [29,34]. Furthermore, Anbarani et al. [32] demonstrated that the protein was located between the starch granules in the continuous phase and reduced the starch rearrangement, which caused gel softening. Previously, the reduction in the starch gel harness after the addition of protein has been reported in several studies [2,9,11]. Cohesiveness indicates the required energy to tolerate the deformation within the food, and the rearrangement of leached amylose plays a significant role in starch gel cohesiveness [24]. The addition of MBPI generally decreased the cohesiveness of NCS/MBPI gels, which suggested that the lower energy is required to deform the NCS gels in the presence of MBPI [32]. Springiness represents the foodstuff’s elasticity [24]. The springiness of NCS gels did not show a regular trend with the addition of MBPI. However, previous studies showed the reduction in springiness value in starch-protein systems by increasing the protein content [20,35]. Gumminess shows the required energy for semi-solid food disintegration [9]. Gumminess of the NCS gels showed the same trend as hardness and decreased with the addition of MBPI, which could be because of the impact of hardness in determining this parameter [24].

### 3.3. Morphological Properties

**Figure 2:**
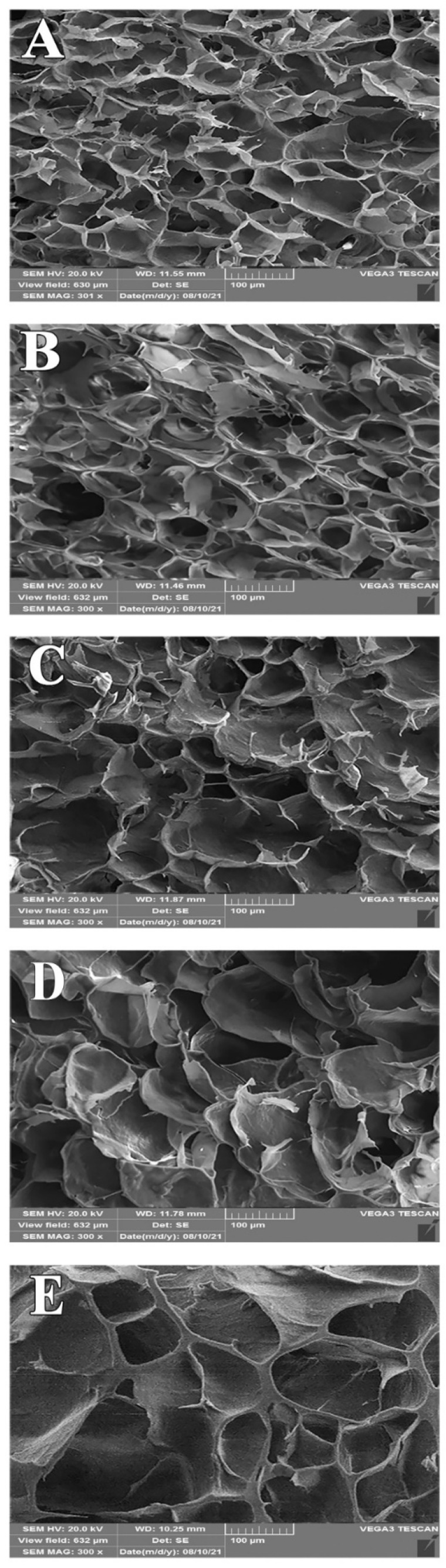
SEM of the NCS/MBPI gels. NCS (**A**), NCS + MBPI 2% (**B**), NCS + MBPI 4% (**C**), NCS + MBPI 6% (**D**), NCS + MBPI 8% (**E**).

The SEM micrographs of the freeze-dried NCS/MBPI gels are shown in Figure 2. The NCS gel (Figure 2A) showed a dense, porous, and honeycomb-liked network structure. This three-dimensional network structure formed due to the swelling and amylose leaching of starch granules into a continuous starch network during the gelatinization [24,36]. The pores of the starch–protein complex gels became larger and thicker, and the structure of the gels became looser with the increasing MBPI concentrates, which was more noticeable in samples containing 8% MBPI (Figure 2E). The larger pore sizes and lower structural cohesion indicate the weakening of the gel structure [8]. These results were consistent with the results of the rheological and textural tests, which showed that the structure of the NCS gel became weaker by the addition of the MBPI. In addition, Joshi et al. [19] and Sun and Xiong [20] reported similar results for starch-protein composites. Moreover, Li et al. [37] observed smaller pores and denser structures in corn starch gels compared to starch-soy protein concentrate composite gels.

### 3.4. XRD Analysis

**Figure 3:**
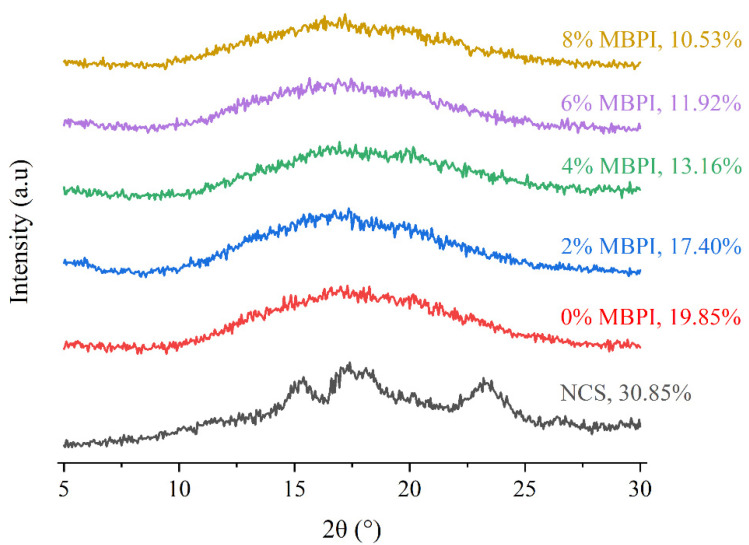
XRD pattern and RC of NCS and freeze-dried NCS/MBPI gels at different MBPI concentrates.

The XRD patterns and RC of samples are presented in Figure 3. The NCS showed the A-type crystalline pattern with strong single diffraction peaks at 15.4° and 23.2° and obvious doublet peaks at 17.4° and 18.1° [38]. After pasting, NCS showed only a single diffraction peak at 17.4°. Compared with NCS, the addition of MBPI did not affect the crystal type but decreased the RC of the starch-protein composites, especially at higher concentrates. These results could indicate that the addition of MBPI disturbs the crystalline region of NCS [39]. Similar results for starch-protein mixtures were shown by Niu et al. [29] and Zhang et al. [40]. Furthermore, Zheng et al. [18] reported that the protein could delay the recrystallisation of amylopectin. Chen et al. [39] demonstrated that the soy protein could reduce the RC of corn starch by absorbing the granule starch moisture and interrupting the crystalline region of starch.

### 3.5. Turbidity Measurements

**Figure 4:**
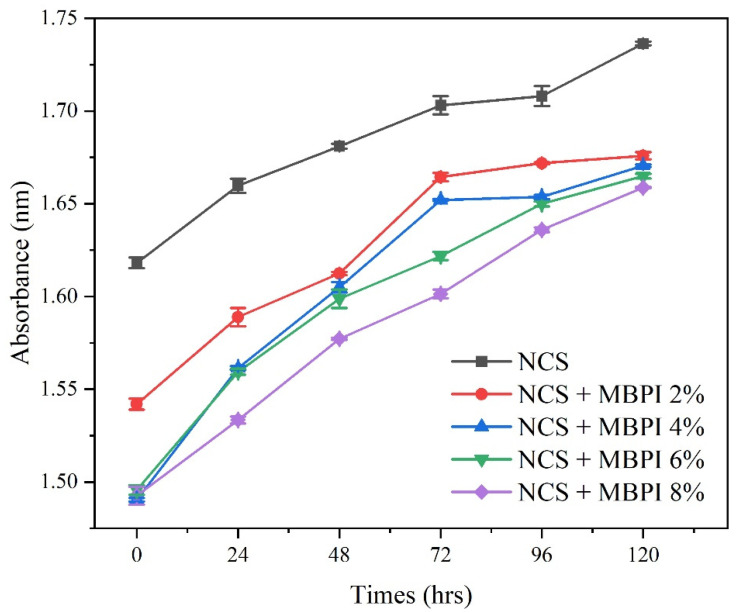
The turbidity of NCS and NCS/MBPI gels at different MBPI concentrates (2%, 4%, 6%, and 8%) during 120 h storage at 4 °C.

The turbidity of NCS and NCS/MBPI gels at different MBPI concentrates are presented in Figure 4. According to Berski et al. [41], turbidity evaluates the early physical changes in gelatinized starch and its retrogradation during storage, which is attributed to the starch molecular rearrangements, especially amylose. The NCS gel had the highest initial turbidity compared to the NCS/MBPI gels. Moreover, for all samples, the turbidity was increased during storage. Previously, Ghumman et al. [42] reported that the albumin could decrease the starch’s turbidity; however, globulin increased it. The reduction in the sample’s turbidity in the present study may be due to the higher ratio of albumin/globulin in the MBPI (0.42), compared to the other legumes proteins, such as lupine (0.16), pea (0.32), and soybean (0.11) [43]. Moreover, the excellent WHC of MBPI could promote the integrity of starch granules and stabilize the starch gels against retrogradation [44]. These observations indicated that the MBPI could retard the retrogradation of the NCS gel, which were in accordance with our previous results. In addition, the clarity of starch-based foodstuffs is an important factor in attracting customers [45]. Therefore, adding the MBPI to starch-based products, in addition to improving its nutritional value and retrogradation properties, can also fulfil the customers’ demands.

### 3.6. Syneresis

**Figure 5:**
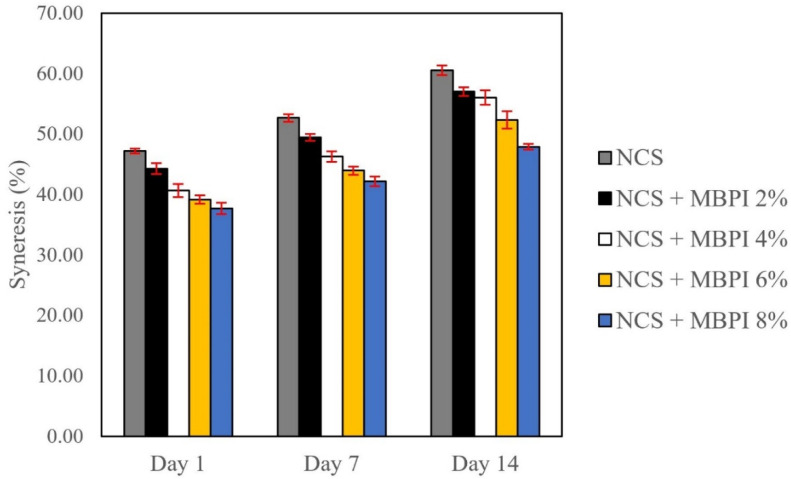
The syneresis of NCS and NCS/MBPI gels during refrigerated storage for 1, 7, and 14 days.

Syneresis is water separation of the system during storage and happens when the leached starch molecules begin to reassociate. Consequently, the gel network shrinks and becomes rigid. This phenomenon occurs along with the reduction in the WHC of the system, which is suggested as the rate of a gel’s retrogradation during refrigerated storage [8,32]. According to Figure 5, the addition of MBPI decreased the syneresis of NCS gels after 1, 7, and 14 days from 47.16–37.98, 52.64–42.13, and 60.53–47.87%, respectively. The reduction in syneresis in the first 7 days could correlate with the amylose reassociation and rapid retrogradation of NCS, while the syneresis after 14 days of storage might indicate the amylopectin rearrangement and long-term retrogradation of NCS [32]. These observations were correlated with previous studies about starch-protein systems. Colombo et al. [36] and Ribotta and Rosell [46] showed that soy protein and peanut protein isolates could reduce the syneresis of corn starch by trapping the water molecules in the system, due to its high-water retention capacity. Moreover, some interactions developed between the leached amylose and amylopectin during the storage, which caused syneresis. The presence of MBPI in the continuous phase of the system and its interaction with leached molecules could retard these interactions and delay the system’s retrogradation. According to the results, addition of MBPI could reduce both short- and long-term retrogradation of NCS, which is important to its usage in starch-based products, such as pudding and custard.

## 4. Conclusions

This study demonstrated that MBPI could act as a lubricant in the NCS/MBPI system and weaken the gel network of NCS by representing the higher sensitivity of rheological parameters to frequency changes, as well as the lower gel firmness and lower structural cohesion after the MBPI addition. In addition, MBPI can interact with leached macromolecules (amylose and amylopectin) and retard the short- and long-retrogradation of the NCS gel. Moreover, MBPI could trap the water molecules in the system because of its high WHC, improve the system’s clarity, reduce the gel’s syneresis, and extend the shelf life of the products. Therefore, the interactions of MBPI and leached macromolecules and water molecules during gelatinization could affect the rheological, textural, and structural properties of NCS gels in different ways. Based on the results of this study, MBPI can be used in starch-based products to reduce the retrogradation, and improve the functional properties.

## Figures and Tables

**Table 1:** Textural properties of NCS and NCS/MBPI gels at different MBPI concentrations.

MBPI (%)	Hardness	Cohesiveness	Springiness	Gumminess
0	209.61 ± 1.31 ^a^	0.953 ± 0.013 ^a^	2.347 ± 0.051 ^b^	199.81 ± 2.99 ^a^
2	201.50 ± 1.41 ^b^	0.920 ± 0.019 ^b^	2.478 ± 0.003 ^a^	185.31 ± 3.97 ^b^
4	189.74 ± 2.07 ^c^	0.887 ± 0.012 ^c^	2.373 ± 0.004 ^b^	168.35 ± 4.00 ^c^
6	162.79 ± 2.06 ^d^	0.869 ± 0.012 ^c^	2.256 ± 0.033 ^c^	141.40 ± 2.50 ^d^
8	150.45 ± 1.91 ^e^	0.881 ± 0.005 ^c^	2.326 ± 0.041 ^b^	132.58 ± 2.40 ^e^

Means in the same column with different letters are significantly different (*p* < 0.05). Means ± standard deviations of triplicate analysis.

## Data Availability

The data presented in this study are available upon request from the corresponding author.
